# Structure and predictive functional profiling of microbial communities in two biotrickling filters treated with continuous/discontinuous waste gases

**DOI:** 10.1186/s13568-018-0726-9

**Published:** 2019-01-04

**Authors:** Rongfang Feng, Meiying Xu, Jianjun Li, Shaobin Huang, Gang Zhao, Xiang Tu, Guoping Sun, Jun Guo

**Affiliations:** 1 School of Bioscience and Bioengineering, South China University of Technology, Guangzhou, 510006 People’s Republic of China; 2 Guangdong Institute of Microbiology, Guangzhou, 510070 People’s Republic of China; 3 State Key Laboratory of Applied Microbiology Southern China, Guangzhou, 510070 People’s Republic of China; 4 Guangdong Provincial Key Laboratory of Microbial Culture Collection and Application, Guangzhou, 510070 People’s Republic of China; 5 School of Environment and Energy, South China University of Technology, Guangzhou, 510006 People’s Republic of China

**Keywords:** Biotrickling filter, Volatile organic compounds (VOCs), Discontinuous operation, Microbial community, PICRUSt

## Abstract

Two biotrickling filters were operated in continuous (BTF1) and discontinuous (BTF2) modes at a constant empty bed residence time of 60 s for 60 days. From day 60, the operation mode of each BTF was oppositely switched. Higher removal efficiencies of five aromatic pollutants were recorded with BTF1 (> 77.2%). The switch in the operation mode did not alter the removal performance of BTF1. Comparatively, BTF2 was not successfully acclimated in the discontinuous operation mode. Once the mode had been switched to continuous mode, the removal efficiencies of BTF2 on all pollutants increased drastically and finally exceeded the values observed in BTF1, with the single exception of *p*-xylene. Principle coordinate analysis and analysis of similarities (ANOSIM) showed that the structure of the microbial communities differed considerably between both BTFs (R = 1.000, *p *< 0.01) as well as before and after the switch in BTF2 (R = 0.996, *p* < 0.01). The random forest model demonstrated that *Mycobacterium*, *Burkholderia*, and *Comamonas* were the three most important bacterial genera contributing to the differences in microbial communities between the two BTFs. Metagenomics inferred by PICUSt (phylogenetic investigation of communities by reconstruction of unobserved states) indicated that BTF2 had high degradation potential for aromatic pollutants, although those genes involved in biofilm formation were less active in BTF2 than those in BTF1.

## Introduction

Volatile organic compounds (VOCs) are generated from many industrial activities such as petroleum refining, coating and furniture manufacturing (Lee et al. 2009; Cheng et al. 2016). The release of VOCs into the atmosphere poses a severe threat to human health and welfare (Gallastegui et al. 2011). Furthermore, many kinds of VOCs are primarily responsible for the formation of photochemical smog (Guo 2012; Wang 2015). Large quantities of waste gases containing VOCs are emitted each year in China, leading to increasingly severe air pollution; in response to this, Chinese authorities are reinforcing the management of pollution control (Tong et al. 2013; Zhu et al. 2016). Benzene and toluene, together with ethyl benzene and xylene constitute BTEX, account for approximately 59% (w/w) of gasoline pollutants (Rahul et al. 2013). BTEX ranked 78 of 275 substances that were identified as showing the most significant potential threat to human health based on the Comprehensive Environmental Response, Compensation and Liability Act (CERCLA 2007). Toluene, ethyl benzene and o-xylene are highly toxic and mutagenic, and many health problems, including kidney failure, heart attack, and liver disease, are likely to be caused by chronic exposure to these substances (Wilbur et al. 2008; Singh et al. 2010; Rahul et al. 2013).

Biofiltration is generally considered a cost-saving and environmentally friendly approach for the abatement of air pollution (Leson and Winer 1991; Nikiema et al. 2007). Many efforts have been made to optimize the configuration of bioreactors to enhance their overall performance (Irvine and Moe 2001; Kim et al. 2005). Effects of various environmental factors such as EBRT, pH, temperature, moisture content, and pressure drop, as well as the microbial communities inside, have been extensively studied (Baquerizo et al. 2009; Cabrol et al. 2012; Pérez et al. 2016; Copelli et al. 2017; Ordaz et al. 2018). These studies were conducted under steady conditions. However, many actual emission sources generate waste gases in a discontinuous mode, and the performance of bioreactors under this unsteady condition has been rarely reported (Lee et al. 2009; Lebrero et al. 2013; San-Valero et al. 2013; Alinejad et al. 2017). According to Qi and Moe (2006), a biofilter operated under discontinuous mode required more time to adapt to the pollutants compared with one that was continuously fed with pollutants. There are still large uncertainties concerning the impact of discontinuous mode on the removal performance, function and composition of microbial communities as well as biofilm formation, especially under discontinuous conditions from the beginning of operation. Studies related to these issues are very scarce in the literature.

The abatement performance of biofiltration reactors relies heavily on the biofilm formed on the surface of packing materials (Acuña et al. 2002). Biofilm formation allows a microbial lifestyle that is entirely different from the planktonic state (Flemming and Wingender 2010). The formation of mature biofilm on various surfaces is a major consideration when running biological reactors in discontinuous mode since some of the attached cells can detach from the surface during the idle phase and return to the planktonic form. In this situation, biofilm formation is extremely difficult. Microorganisms differ greatly in their ability to form biofilm or to degrade pollutants (Lee et al. 2009; Alinejad et al. 2017). Some types of microorganisms may play a leading role in biofilm formation, whereas others possess strong degrading capabilities but have a weak aptitude for forming biofilm. Therefore, the understanding of the specific functions of different microorganisms will provide us with insight into the biofiltration process of VOC-containing waste gases during discontinuous operation mode.

In this study, we used two biotrickling filters fed with a mixture of five aromatic compounds in continuous and discontinuous modes. The removal efficiencies of each bioreactor were compared for five aromatic compounds. High-throughput sequencing of 16S rRNA genes was employed to further understand the effect of the operation mode on the composition and ecological function of the microbial communities. The structure of microbial communities was compared before and after the switch of operation mode, and important species that contributed to the differences between continuous and discontinuous conditions were identified using the random forest classifier (Breiman 2001). The functions of the microbial communities were also predicted using the phylogenetic investigation of communities by the reconstruction of unobserved states (PICRUSt) (Langille et al. 2013). We aimed to identify the specific microorganisms capable of adapting adverse condition, and then reveal the mechanisms of biofilm formation under stress conditions.

## Materials and methods

### Chemicals

Toluene, ethyl benzene, *p*-xylene, *m*-xylene, and *o*-xylene were all purchased from Aladdin Reagent Co., Ltd. (Shanghai, China) with purities of 99.5%, 99.5%, 99.0%, 99.0%, 98.0%, respectively.

### Experimental set-up

**Fig. 1:**
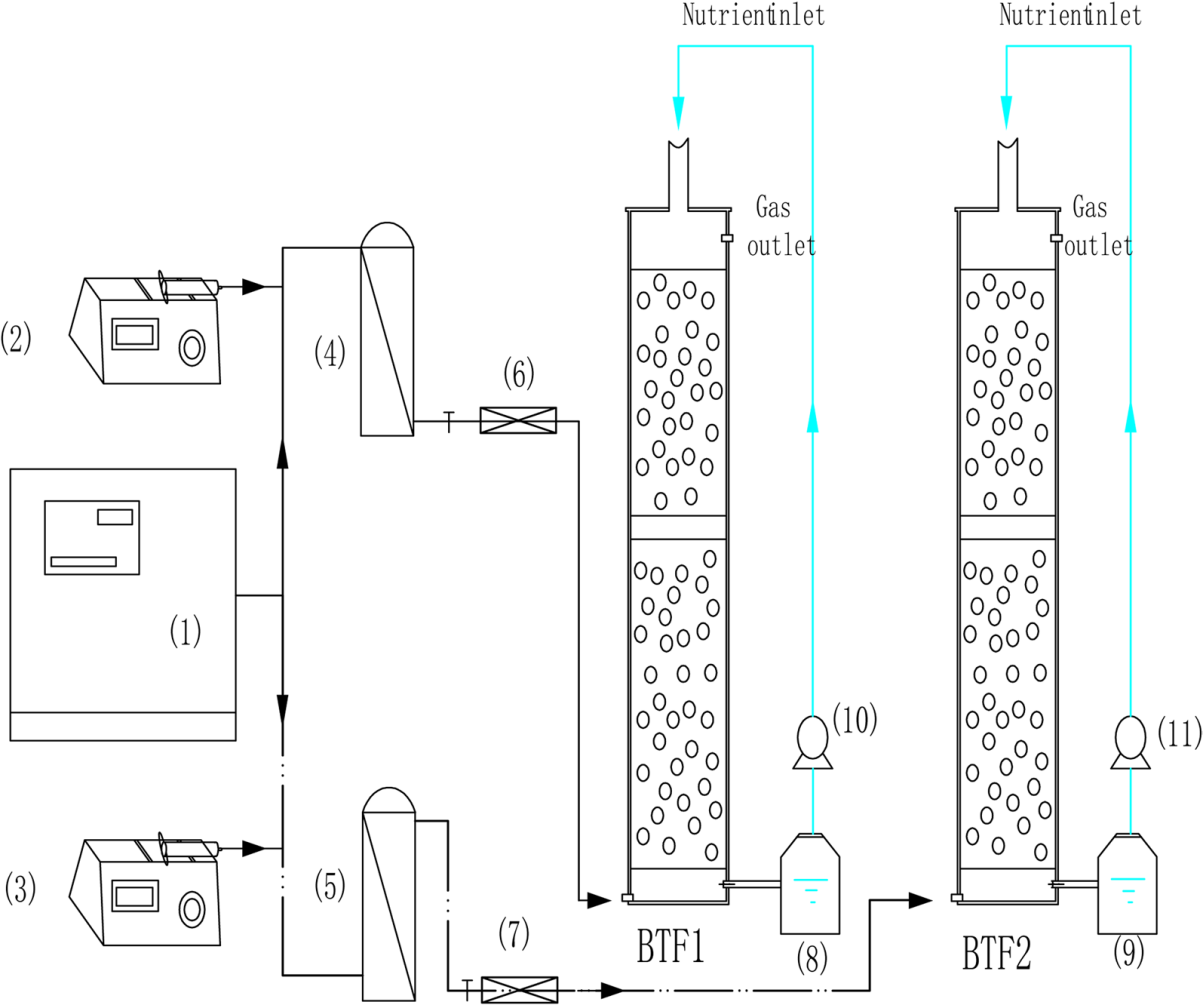
The schematic of BTFs. (1) Air compressor, (2) (3) syringe pump, (4) (5) mixing chamber, (6) (7) three-way valve, (8) (9) nutrient-holding tank, (10) (11) peristaltic pump

The two identical laboratory-scale BTFs employed in this study were made of plexiglass and were designated BTF1 and BTF2. The schematic of the experimental setup is shown in Fig. 1. Each BTF was composed of two columns in a stacked configuration, with an inner diameter of 10 cm and a total height of 38 cm. The resultant packing volume of each BTF was approximately 3 L. Ceramics, with particle sizes of 6–10 mm and a bulk density of 0.57 g/cm^3^, were used as packing materials. To generate the waste gases, compressed air was split into a major and minor airstream. A liquid VOC mixture was injected into the minor airstream via a syringe pump and was finally mixed with the major airstream in a chamber. The mixed VOC vapor was fed from the bottom of both BTFs in the up-flow mode. The desired concentration was obtained by setting the injection rate of the mixed liquid VOCs. Three microbial consortiums enriched from petroleum polluted soil were proved with high capability of degrading toluene, xylene and styrene, respectively. They were acclimated with toluene, xylene and styrene as sole carbon source, respectively, over 2 years in our laboratory. Consortiums acclimated with toluene, xylene and styrene were mixed (1:1:1 v/v/v), and total 300 mL of the mixed culture was then mixed with 3.0 L of nutrient solutions. Each BTF was inoculated with 1.5 L consortium mixture.

### Operation condition

Both BTFs were operated in parallel for 90 days. To evaluate the effect of the operation mode on the BTF’s performance, BTF1 was fed continuously (24 h/day, 7 days/week) with the VOC mixture, while BTF2 was fed discontinuously (8 h/day, 5 days/week) during the first stage (day 0–60). At the beginning of the second stage (day 61–90), the operation mode of BTF1 was switched from continuous to discontinuous. Conversely, BTF2 was switched from the discontinuous to the continuous operation mode.

The VOC mixture was composed of five aromatic compounds including toluene, ethyl benzene, *p*-xylene, *m*-xylene and *o*-xylene (Table 1). From day 37, concentrations of the three xylene isomers were reduced by approximately 50% to avoid possible inhibitive effects. Both BTFs were operated at an EBRT of 60 s, and the trickling rate of nutrient solution was set at 27.7 mL/min. During the idle phase, without the feeding of any VOCs, the BTF was supplied with fresh air and nutrient solution at the same inlet loading and velocity as during the fed phase. A total of 1.5 L of the nutrient solution was drawn off and renewed approximately once every 2 weeks to ensure that there was sufficient nutrients and moisture for the growth of the microorganisms. The nutrient solution contained the following chemical compounds (per liter of water): NH_4_Cl 2.5 g, Na_2_HPO_4_ 1.0 g, KH_2_PO_4_ 0.7 g, MgSO_4_ 0.05 g, CaCl_2_ 0.015 g.

**Table 1:** Compounds used in the experiment and their inlet concentrations

Stage	Days	Inlet concentration (mg/m^3^)				
		Toluene	Ethyl benzene	*p*-Xylene	*m*-Xylene	*o*-Xylene
I^a^	1–36	160	100	100	130	90
	37–60	160	100	50	60	50
II^b^	61–90	160	100	50	60	50

^a^BTF1 was operated in continuous mode, while BTF2 in discontinuous mode

^b^Operation mode of each BTF oppositely switched

### Gas chromatograph analysis

BTF2 was supplied with VOCs beginning at 8:30 on workdays, and gas samples from the inlet and outlet of the two BTFs were collected at 9:30. Gas samples were collected using 2 L Tedlar bags and measured immediately by a gas chromatograph (GC, SHIMADZU GC-2010 plus, Japan) equipped with a capillary column (HP-INNOWAX, 30 m × 0.25 µm × 0.50 mm) and with a flame ionization detector. Nitrogen was used as the carrier gas. The temperatures of the injector and detector were 200 and 250 °C, respectively. The GC oven temperature was programmed as follows: an initial temperature of 35 °C for 3 min, which was increased to 180 °C at 12 °C/min and maintained for 1 min.

To evaluate the performance of the two BTFs, the removal efficiency (RE) for each pollutant was calculated using Eq. (1):

1 $$\text{RE} \; (\%) = ({{\text{C}}_{\text{in}}}-{{\text{C}}_{\text{out}}}/ {{\text{C}}_{\text{in}}} \times 100$$

where C_in_ is the inlet concentration (mg/m^3^), and C_out_ is the outlet concentration (mg/m^3^).

### DNA extraction and high-throughput sequencing

A total of 2 g of packing materials was collected aseptically, in triplicate, from the top and bottom filter layer of each BTF on days 30, 60, 75, and 90. The packing materials were mixed with 10 mL phosphate buffer solution (NaCl 8 g/L, KCl 0.2 g/L, Na_2_HPO_4_ 1.42 g/L, KH_2_PO_4_ 0.27 g/L). After gently shaking, the biofilms were completely detached from the surface by ultrasonication for 10 min. The suspensions were then collected and centrifuged for 10 min at 10,000*g*. The pellet was collected for the DNA extraction.

Genomic DNA was extracted using the MO-BIO PowerSoil^®^ DNA Isolation Kit (MO BIO Laboratories, Inc.) according to the manufacturer’s instructions. Universal primers 515F (5′-GTG CCA GCM GCCGCG GTA A-3′) and 909R (5′- CCC CGY CAA TTC MTT TRA GT -3′) with 12 nt unique barcodes were used to amplify the V4 and V5 hypervariable regions of the 16S rRNA gene for pyrosequencing using a MiSeq sequencer.

### Measurement of biomass and extracellular polymeric substances (EPS)

Triplicates of biofilm materials from 3 g (wet weight) of the packing materials were collected from each layer of the BTFs. The materials were gently washed twice with phosphate buffer and then dissolved in 4 mL of buffer. After being vortexed for 5 min, the materials were subjected to ultrasonic treatment for 10 min. After the detachment of the biofilms, the ceramics were discarded. Nine hundred microliters of the biofilm-containing liquid were removed to measure the total protein content (represented as biomass), and another 1.5 mL of the liquid was used to extract EPS. The 1.5 mL suspensions were centrifuged at 14,000*g* for 20 min at 4 °C, and the supernatant was then ready to measure the content of proteins and polysaccharides in the EPS (Zhu et al. 2012). The total protein content and protein in the EPS was determined by the Bradford assay with bovine serum albumin as the standard (Bradford 1976), and the exopolysaccharide content was measured by the anthrone-sulfuric acid method with glucose as the standard (Dubois et al. 1956).

### Data analysis

The raw data were initially quality-filtered with QIIME Pipeline (Version 1.7.0) (http://qiime.org/tutorials/tutorial.html) to remove readings that did not meet the desired quality. All sequence readings were trimmed and assigned to the corresponding samples based on their barcodes. Multiple steps were taken to trim the sequences, such as the removal of sequences < 200, and requiring an average base quality score of Q < 25. Any chimeric sequences were identified and removed using the Uchime algorithm. The sequences were clustered into operational taxonomic units (OTUs) using a 97% identity threshold. The taxonomy of each 16S rRNA gene sequence was analyzed using the RDP classifier at a confidence level of 80%. To compare the communities between samples, the sequence dataset was randomly subsampled to an equal number of sequences to minimize the impact of the varied sequencing depth.

To directly visualize the difference between samples, principal coordinate analysis (PCoA) was performed using the vegan and ggplot2 packages in R software. Analysis of similarity (ANOSIM) was conducted to test the significance of differences between groups using the vegan package in R software. A random forest model based on the relative abundance of OTUs was employed to further describe the changes in the microbial communities between different running stages (Breiman 2001). Random forest analysis was conducted using the randomForest package in R software based on samples collected from day 60 and day 90. The numbers of trees and random variables per branch were set at 10,000 and 20, respectively. To check the differences in the function of the microbial communities between samples, before and after the switch of the BTF operation mode, the metagenomic content of the samples was inferred from 16S rRNA sequencing data using phylogenetic investigation of communities by the reconstruction of unobserved states (PICRUSt) (Langille et al. 2013).

## Results

### Effects of operation mode on the removal performance of biotrickling filters

**Fig. 2:**
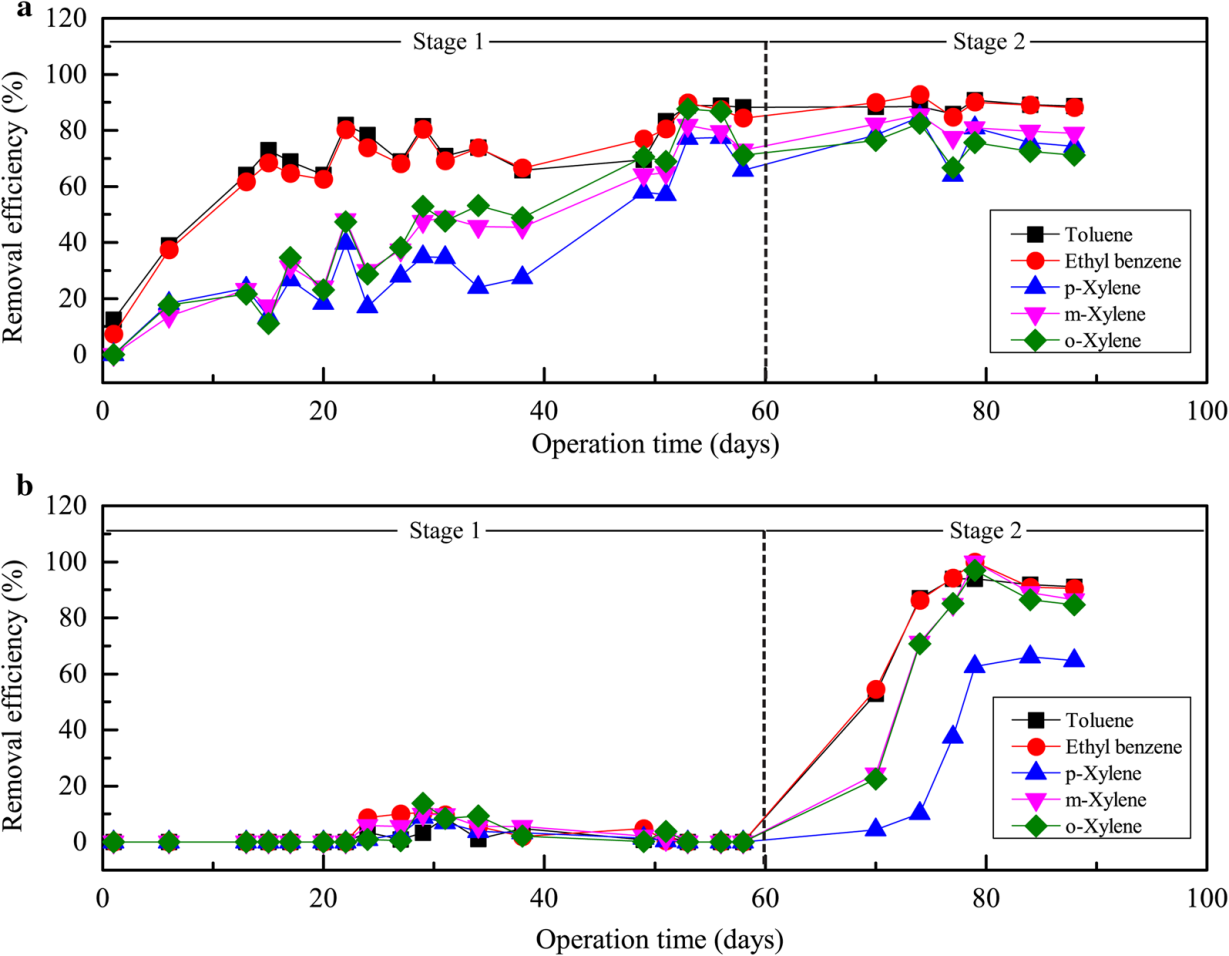
Removal efficiencies of the five aromatic compounds in BTF1 (**a**) and BTF2 (**b**)

During the first experimental stage, BTF1 was fed with the VOC mixture continuously, while BTF2 was fed only 8 h/day and 5 days/week. The removal performance of the two BTFs was compared in terms of removal efficiencies (Fig. 2). The results showed that REs for all aromatic compounds gradually increased in BTF1 after startup. Toluene was among the most efficiently removed compounds in BTF1, followed by ethyl benzene. REs of these two compounds in BTF1 rapidly reached 64.1% and 61.5% on day 13. Comparatively, the three xylene isomers were more difficult to be remove in BTF1, despite the fact that their inlet concentrations were relatively less than the other two pollutants. REs for *p*-xylene, *m*-xylene and *o*-xylene on day 13 were only 23.8%, 23.3% and 21.6%, respectively. After their concentrations were reduced by up to 50% from day 37, an obvious increase in RE was observed not only for each xylene isomer but also for toluene and ethyl benzene. On day 53, REs for toluene, ethyl benzene, *p*-xylene, *m*-xylene and *o*-xylene reached 88.8%, 89.8%, 77.2%, 81.8%, and 87.6%, respectively. Therefore, it could be inferred that the presence of xylene at higher concentrations inhibited the removal of all components in the mixture.

In comparison, the removal performance of BTF2 was extremely poor during the first experimental stage. Removal efficiencies for all pollutants were near zero. It is evident that the discontinuous operation mode severely affected the acclimation of BTF2. The determination of biomass showed that the biomass that accumulated in the bottom layer of BTF2 on day 60 was only 1.75 μg-protein/g-dry packings. This value was considerably less than that seen in the bottom layer of BTF1 on day 60 (85.07 μg-protein/g-dry packings), indicating that the biomass accumulation on the surfaces of packing materials under the discontinuous operation mode was very limited (Table 2). On day 60, the EPS content in the bottom layer of BTF1 was 45.66 μg/g-dry packings, which is one hundred times higher than that found in BTF2 (Table 2). The results showed that the amounts of microbial cells and the capacity of EPS secretion was extremely restricted under discontinuous operation conditions. Both proteins and exopolysaccharides, as the two dominant components of EPS, differed significantly between the two BTFs. At the end of the first stage, the protein content of the EPS was 16.99 μg/g-dry packings and 22.45 μg/g-dry packings in the upper layer and the bottom layer of BTF1, respectively, however, we failed to detect any protein in either layer of BTF2. The exopolysaccharide contents in the EPS reached approximately 14.69 μg/g-dry packings and 23.22 μg/g-dry packings in the upper layer and the bottom layer of BTF1, respectively, but reached only 1.19 μg/g-dry packings and 0.43 μg/g-dry packings in the upper layer and bottom layer of BTF2, respectively. This finding showed that a small number of cells resided in the small amount of EPS in BTF2, explaining the extremely poor performance under discontinuous operation mode.

**Table 2:** Biomass and EPS content during the operating days

Content (μg/g-dry packings)	Time (day)	BTF1		BTF2	
		Upper	Bottom	Upper	Bottom
Proteins	15	44.63 ± 4.43	57.11 ± 2.00	37.39 ± 3.32	25.62 ± 2.16
	30	34.38 ± 2.71	61.58 ± 6.78	13.07 ± 5.79	6.92 ± 0.66
	60	54.78 ± 1.38	85.07 ± 2.98	3.99 ± 3.10	1.75 ± 0.30
	75	53.80 ± 1.81	61.69 ± 1.27	31.74 ± 1.07	28.34 ± 2.01
	90	185.08 ± 8.91	115.54 ± 5.40	78.47 ± 2.00	98.67 ± 3.92
Extracellular proteins	15	11.21 ± 0.69	28.18 ± 1.58	2.68 ± 0.25	0.44 ± 0.09
	30	5.72 ± 0.58	2.81 ± 0.26	0.00 ± 0.00	0.00 ± 0.00
	60	16.99 ± 2.26	22.45 ± 1.51	0.00 ± 0.00	0.00 ± 0.00
	75	15.43 ± 0.67	19.58 ± 0.29	1.21 ± 0.21	2.72 ± 0.56
	90	29.43 ± 0.50	18.46 ± 0.79	0.00 ± 0.00	3.38 ± 0.09
Exopolysaccharides	15	0.35 ± 0.07	3.20 ± 0.17	0.00 ± 0.00	0.00 ± 0.00
	30	5.63 ± 1.19	19.45 ± 1.3	0.77 ± 0.39	0.00 ± 0.00
	60	14.69 ± 0.47	23.22 ± 1.25	1.19 ± 0.34	0.43 ± 0.11
	75	9.35 ± 0.29	13.74 ± 0.32	1.63 ± 0.18	3.29 ± 0.26
	90	8.33 ± 0.48	11.52 ± 1.20	0.00 ± 0.00	27.27 ± 1.30
EPS	15	11.56 ± 0.63	31.39 ± 1.74	2.68 ± 0.25	0.44 ± 0.09
	30	11.35 ± 1.06	22.26 ± 1.54	0.77 ± 0.39	0.00 ± 0.00
	60	31.67 ± 2.04	45.66 ± 2.71	1.19 ± 0.34	0.43 ± 0.11
	75	24.78 ± 0.87	33.32 ± 0.59	2.84 ± 0.05	6.01 ± 0.72
	90	37.76 ± 0.43	29.98±1.88	0.00 ± 0.00	30.65 ± 1.36

### Removal behavior of biotrickling filters after the switch of operation mode

From day 61, the operation mode of each of the BTFs was switched. The switching of the operation modes did not significantly decrease the removal performance of BTF1. Compared with day 58, on day 88, REs for toluene and *o*-xylene remained at the same level. REs for ethyl benzene, *p*-xylene, and *m*-xylene increased slightly by 4.9%, 8.7% and 6.0%, respectively.

Once the operation mode was switched on day 61, REs for all compounds increased drastically in BTF2. The overall performance of BTF2 was actually superior to that of BTF1. On day 88, REs for all the compounds exceeded values found in BTF1. The only exception was *p*-xylene, which was removed in BTF2 at 64.7% RE. The amount of biomass in BTF2 increased rapidly after the switch and reached 78.47 μg-protein/g-dry packings and 98.67 μg-protein/g-dry packings in the upper layer and the bottom layer, respectively, of BTF2 on day 90 (Table 2). Furthermore, the EPS content in the bottom layer increased significantly (*p *= 0.0000 < 0.01) once BTF2 was switched into continuous loading, without showing a significant difference compared to the EPS contents in the bottom layer of BTF1 (*p *= 0.6468 > 0.05). This result showed that biofilm was formed successfully in the bottom layer once the operation mode was switched to continuous, at least partially explaining the increase in removal performance in BTF2. However, the EPSs content in the upper layer was near zero, indicating that the microorganisms colonized in the upper layer mainly existed in the form of planktonic cells.

### Links between the structure of microbial communities and removal performance of biotrickling filters

All 16S rRNA genes obtained by high-throughput sequencing from a total of 51 samples were deposited in the NCBI Sequence Read Archive (SRA) database (Accession no. SRP123827). This database generated an average of 22,511 quality sequence reads for each sample. After subsampling, 6000 sequence reads were obtained for each sample. A total of 3866 operational taxonomic units (OTUs, grouped at 97% sequencing similarity) were obtained for all samples and ranged from 75 to 715 OTUs per individual sample. Approximately 95% of the OTUs in BTF1 and 84% of the OTUs in BTF2 could be classified into six bacterial phyla, including *Proteobacteria*, *Actinobacteria*, *Bacteroidetes*, *Acidobacteria*, *Planctomycetes* and *Cyanobacteria*. *Proteobacteria* was among the most predominant bacterial phyla in BTF1. Its relative abundance in BTF1 varied from 70.9 to 97.0%. The phylum *Proteobacteria* also consistently predominated in BTF2. However, its relative abundance in BTF2 gradually decreased from 51.3% on day 60 to 14.8% on day 90, which coincided with an increase in the phylum *Actinobacteria* (from 31.2 to 47.4%). The phylum *Proteobacteria* primarily comprises the classes *Betaproteobacteria*, *Alphaproteobacteria* and *Gammaproteobacteria*. Among them, the class *Betaproteobacteria* accounted for 88.1% of all the sequences belonging to *Proteobacteria* in BTF1 and 67.1% in BTF2.

**Fig. 3:**
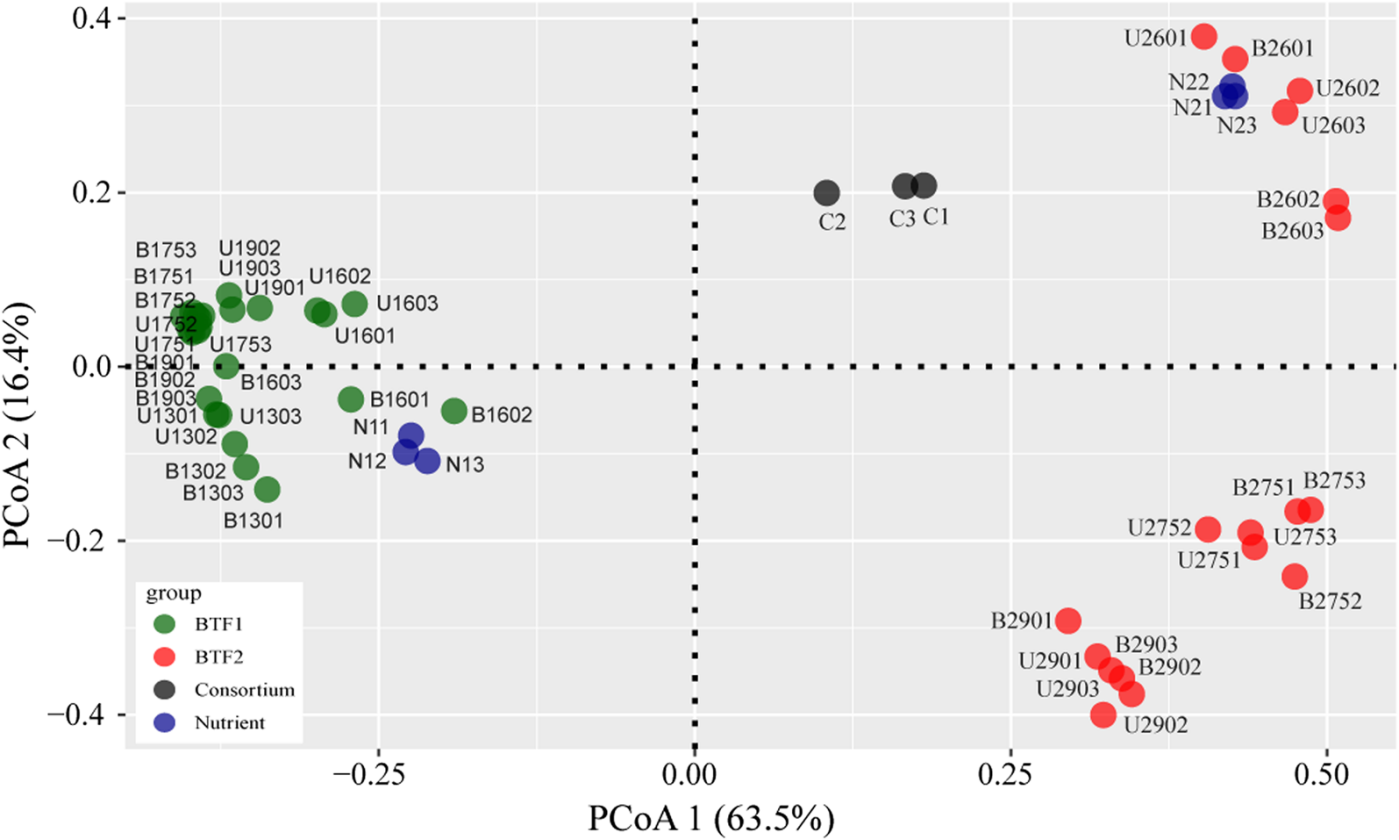
Principal coordinate analysis (PCoA) of microbial communities. Samples collected from the bottom layer and upper layer are labeled by letter respectively. First number denote BTF1 or BTF2. Last two numbers indicates the sampling day. Sample from initial inoculums are showed with black circles, and sample from recycling nutrient showed with blue circles

To show the shifts in the microbial communities with operation time, weighted principal coordinate analysis was performed using the OTU dataset based on the Bray-Curtis dissimilarity. The results showed that a total of 79.9% variance in the data could be explained by the first two principal coordinate axes. Figure 3 shows that the samples from BTF1 and BTF2 were clustered into two distinctly different groups along the PCoA 1. Analysis of similarities (ANOSIM) also showed that the structure of microbial communities differed considerably between the two BTFs (R = 1.000, *p *< 0.01). This finding means that the operation mode has a large impact on the structure of the microbial community. In addition, the microbial communities in BTF2 changed more dynamically than those in BTF1. ANOSIM showed that microbial communities in BTF2 differed highly between groups before and after the switch (R = 0.996, *p *< 0.01). Compared to the microbial communities of BTF2, those of BTF1 were robust throughout the experiment, and no significant difference was observed between communities before or after the switch (R = 0.519, *p* < 0.01).

### Core microorganisms revealed by random forest model

An attempt was made to link microbial species to their ecological function in pollutant removal and biofilm formation. Significantly different OTUs between the two BTFs were identified by constructing a random forest classification model based on the dataset of the OTUs with more than 0.1% relative abundance, which accounted for 98.0% of all sequencing reads in BTF1 and 93.5% in BTF2. The out-of-bag error rate of the classifier was 0.0%, suggesting that the classifier performed better with higher predictive accuracies. OTUs with higher importance scores in the random forest model and their relative abundance are listed in Table 3. These OTUs were assigned to several bacterial genera, including *Mycobacterium*, *Burkholderia*, *Comamonas*, *Sphingomonas*, *Aquisphaera*, and *Nitrosospira*. Among these, *Mycobacterium* was the most important genus contributing to the divergence of microbial communities between BTF1 and BTF2. Members of this genus were highly abundant in BTF2 with an average relative abundance of 32.5%, which is notably higher than that in BTF1 (2.2%). After the switch of operation modes, the relative abundance further increased from 25.8 to 37.3%. Considering the substantial increase in the removal performance of BTF2, it can be inferred that this core species is an important degrader of pollutants in BTF2. The genus *Burkholderia*, in contrast, consistently predominated in BTF1. Its average relative abundance in BTF1 reached up to 73.2%, which is far higher than that in BTF2 (4.9%). However, the genus *Burkholderia* was abundant in BTF2 when the operation mode was switched. Its relative abundance increased from 0.7 to 7.1% after the switch. Therefore, the members of the genus *Burkholderia* could be considered other key degraders of pollutants in both BTFs.

**Table 3:** OTUs with high importance score in the random forest model

OTUs	Genus	Mean decrease accuracy	Mean decrease Gini	Relative abundances (%)			
				BTF1 on day 60th	BTF1 on day 90th	BTF2 on day 60th	BTF2 on day 90th
denovo38658	*Mycobacterium*	22.04	0.95	2.56	1.88	25.71	37.29
denovo36542	*Burkholderia*	21.96	0.91	69.68	79.37	0.71	7.05
denovo13413	*Comamonas*	21.39	0.89	0.02	0.02	25.20	2.55
denovo41479	Unclassified	17.51	0.59	0.31	0.38	0.01	0.00
denovo23573	*Sphingomonas*	16.16	0.53	1.89	1.84	0.01	0.00
denovo48893	Unclassified	13.97	0.35	0.01	0.01	2.23	0.89
denovo35334	*Aquisphaera*	12.69	0.29	0.18	0.31	0.18	0.17
denovo48332	*Nitrosospira*	12.15	0.28	0.00	0.00	3.34	0.24
denovo34336	*Rhodanobacter*	12.00	0.25	0.01	0.04	9.83	3.93
denovo38117	*Gp*3	8.19	0.14	4.60	2.23	0.01	0.00
denovo18013	Unclassified	7.29	0.10	0.34	0.27	0.00	0.00
denovo24085	*Microbacterium*	6.93	0.04	0.01	0.09	1.29	0.64
denovo26479	Unclassified	6.74	0.04	0.20	0.27	3.49	4.25
denovo1622	*Candidimonas*	6.55	0.05	0.01	0.02	0.13	0.26
denovo34467	Unclassified	6.16	0.05	0.00	0.00	0.92	0.25
denovo3373	Unclassified	5.79	0.05	0.00	0.00	2.89	0.23
denovo42748	*Enterobacter*	5.15	0.05	0.02	0.00	0.03	0.13
denovo13263	*Chryseobacterium*	4.14	0.01	0.02	1.44	0.00	0.00
denovo8121	Unclassified	4.07	0.02	6.22	0.08	1.90	20.67
denovo17967	*Nakamurella*	4.05	0.01	0.00	0.00	0.06	0.01
denovo25007	*Pseudomonas*	3.98	0.01	0.00	0.00	0.01	0.25
denovo10012	Unclassified	3.48	0.02	0.80	0.14	0.00	0.00
denovo9612	Unclassified	3.22	0.01	0.00	0.18	4.37	0.08
denovo48474	Unclassified	2.64	0.02	0.35	0.00	0.04	5.05
denovo13873	*Hydrotalea*	2.56	0.01	0.11	0.61	0.01	0.01
denovo20924	*Acidocella*	2.36	0.01	3.65	0.14	0.00	1.13
denovo29433	*Rhodopseudomonas*	1.88	0.00	1.34	3.50	2.48	0.87

*Comamonas* was the third important genus contributing to the divergence between BTF1 and BTF2, which predominated in BTF2. However, its relative abundance decreased substantially from 25.2 to 2.6% after the switch. This genus may be more active or competitive under oligotrophic conditions. Additionally, OTUs 41,479, 38,117, and 18,013, as well as members of the genus *Sphingomonas*, predominated in BTF1, but fewer occurred in BTF2. In contrast, OTUs 18,893, 26,497, and 3373, as well as members of the genera *Nitrosospira* and *Rhodanobacter*, predominated in BTF2 but were scarce in BTF1. It is worth noting that OTUs 8121 and 48,474 represented microbial species, similar to the genus *Mycobacterium*, which were abundant in BTF2 only after the operation mode was switched. Their relative abundances greatly increased from 1.9 to 20.7% and from 0.04 to 5.1% in BTF2 after the switch, which coincided with a significant decrease in BTF1.

### Gene families involving biofilm formation and aromatic compounds degradation

**Fig. 4:**
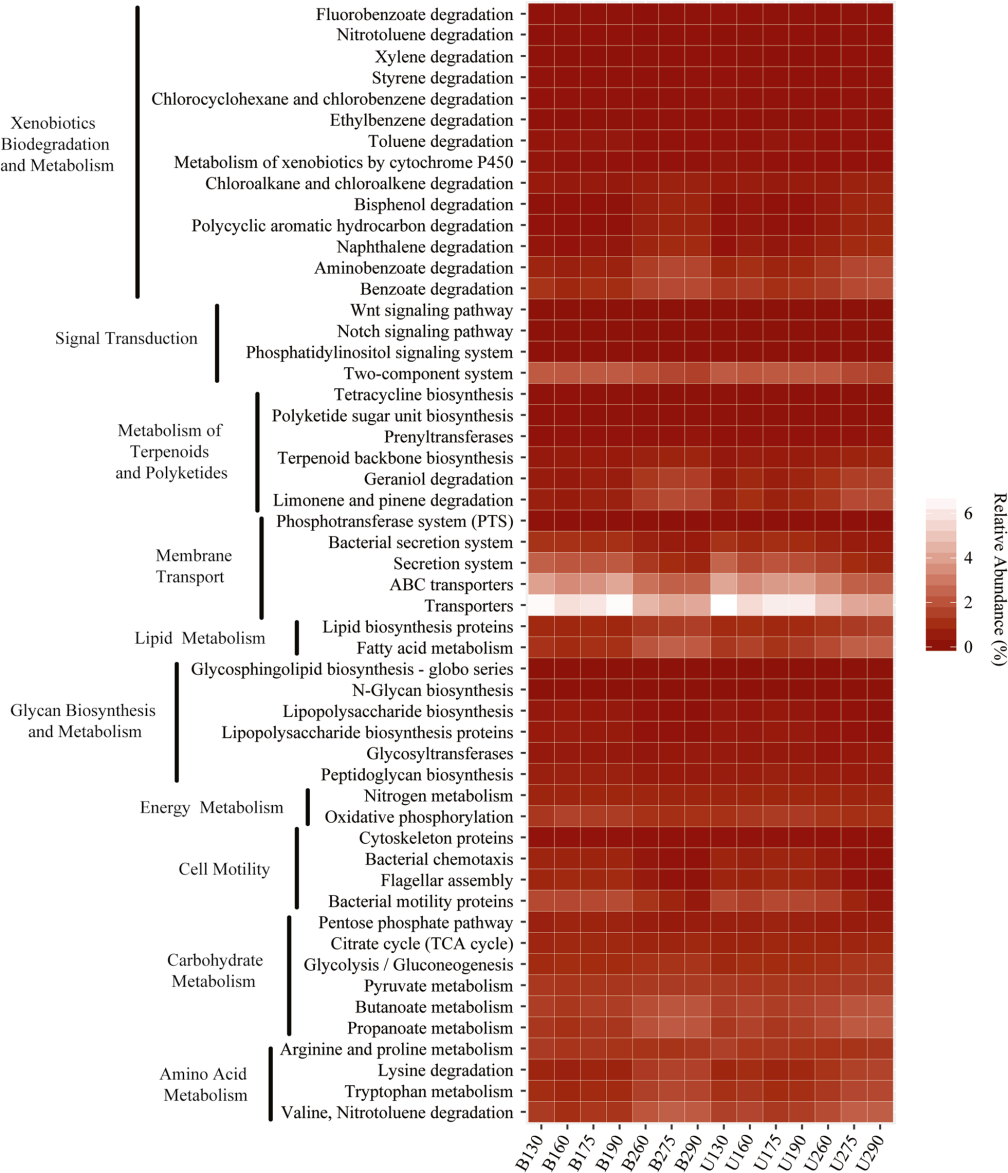
Heatmap showed the changes of top predictive gene families with operation mode in the two BTFs

The shifts in microbial communities with the change in operation mode were described in the above sections. To understand the functional meanings of these shifts during BTF operation, the metagenomic content of all samples was inferred from 16S rRNA gene sequence data using PICRUSt. A total of 328 functional gene families were inferred. A heatmap was drawn to display the changes in the top 53 functional genes (Fig. 4). The results showed that transporters and ABC transporters were among the two most abundant gene families in both BTFs. The relative abundances of the two gene families were significantly higher in BTF1 than those in BTF2 across the experiment. For instance, transporter-encoding gene families predominated in the bottom layer of BTF1 (5.79%) and BTF2 (4.58%) on day 60. After the switch of operation mode, the relative abundance of this gene family was still much higher in BTF1 (6.75%) than that in BTF2 (4.21%). ABC transporter-encoding gene families showed a similar change trend. Their relative abundance was 3.42% in BTF1 and 2.77% in BTF2 on day 60 and 4.14% in BTF1 and 2.37% in BTF2 on day 90. Gene families involving two-component systems, which are responsible for signal transduction, were also prevalent in BTF1 and BTF2. Similarly, two-component signal transduction system-related genes dominated in BTF1 compared to those in BTF2 regardless of operating times (2.10% vs 1.88% on day 60; 2.19% vs 1.49% on day 90).

Moreover, the relative abundances of gene families involved in cell motility such as flagellum assembly, cytoskeleton proteins, and chemotaxis were also lower in BTF2 than those in BTF1. After the switch of operation mode, the relative abundance of these gene families increased slightly in BTF1 and significantly decreased in BTF2 (*p* < 0.05). It is worth noting that, before the switch, the total relative abundances of the genes encoding enzymes for xenobiotic biodegradation, such as those for benzoate, aminobenzoate, toluene, styrene, and xylene, were higher in the bottom layer of BTF2 than their counterpart values in BTF1 (8.1% vs 4.5%). This suggests that BTF2 in fact harbored microbial species with high degradation capabilities for these aromatic compounds.

## Discussion

Five aromatic compounds were used as target pollutants in this study. Our findings demonstrated that toluene and ethyl benzene could be easily removed in the continuously operated BTF1. However, the presence of xylene inhibited the overall removal performance of BTF1. The decrease in the inlet concentrations of xylene reduced this inhibitory effect. Interactions between the components of mixed gases have been reported in the literature, but some reports were conflicting. For instance, Strauss et al. (2004) and Kim et al. (2009) reported that the removal of toluene was inhibited by *p*-xylene, while the removal of *p*-xylene was enhanced by toluene. Another study showed that xylene isomers decreased the RE of toluene, but the effect of toluene on xylene was negligible (Jorio et al. 1998). The inhibitory effect between these aromatic compounds was explained by their similarities in the chemical structure, the metabolic pathways and the enzymes (Gallastegui et al. 2011). However, the interaction during the removal of similar compounds was also influenced by other factors such as the inlet loading rates, the type of microorganisms and the configuration of the bioreactor.

Many industrial waste gases are emitted discontinuously. The purification of discontinuously emitted waste gases by biofiltration still remains challenging. Our findings suggest that BTF2 cannot be acclimated and cannot form a robust biofilm successfully under discontinuous operation mode. Biomass accumulation inside BTF2 was approximately 47 times lower than that observed in the continuously operated BTF1. EPS, which is the key component of biofilm, was approximately one hundred times higher in BTF1 than that in BTF2, indicating that the biofilm formation in BTF2 was severely affected. Under discontinuous operation mode, microbial cells usually enter a so-called endogenous metabolic state during idle times due to the insufficiency of carbon sources. The growth rates of microorganisms were thus lowered (Gallastegui et al. 2011). Additionally, biofilm formation is a highly complex process involving several steps, such as reversible adhesion to solid surfaces, irreversible attachment and EPS production (Molin and Tolker-Nielsen 2003; Zoubos et al. 2012). Those microbial cells reversibly adhered to the surface of packing materials can easily detach from the surface when nutrients are limited. In this situation, it is difficult to increase the density of microbial cells to a threshold level at which quorum sensing signal molecules can be produced (Kim et al. 2014).

The switch in the operation mode from continuous to discontinuous did not decrease the removal performance of BTF1, indicating that once enough biomass had accumulated or a mature biofilm had formed, its robustness was highly enhanced. In a well-organized biofilm, microbial cells aggregate closely with each other and embed in a sticky matrix that is primarily composed of extracellular polymeric substances (EPS). Microorganisms inside the biofilm can coordinate their behaviors synchronously when environmental conditions change (Cheng et al. 2016). Furthermore, with the protective effects provided by EPS, the resistance of microbial cells to various adverse conditions can be greatly enhanced (Dang and Lovell 2000; Jung et al. 2013). However, the microorganisms in the bottom layer would seriously suffer from the starvation shock in some extent, and need time to readapt to the changed environmental condition, leading to relative lower biomass than in the upper layer.

The switch in the operation mode from discontinuous to continuous greatly enhanced the removal performance of BTF2 and promoted the accumulation of biomass as well as the production of EPS in the bottom layer, suggesting that continuous operation mode stimulated the growth of microorganisms and the formation of biofilm in BTF2. It can be inferred that BTF2 must have harbored some kinds of microorganisms that potentially had the capability to degrade aromatic compounds. These microorganisms could not proliferate under the discontinuous operation mode due to the limitation of carbon sources. When continuously fed VOCs were loaded into BTF2 from the bottom, the numbers of these microorganisms adhered on the packing materials of the bottom layer increased rapidly to the threshold at which a biofilm began to form. It is well known that microbes rely heavily on quorum sensing systems to form biofilms (Rohwerder et al. 2003). As mentioned above, although members of the genus *Comamonas* predominated in BTF2, their growth rate was too slow to reach a sufficient number of cells to form a biofilm.

The analysis of high-throughput sequencing data revealed that the two BTFs formed very differently structured microbial communities. The operation mode had strong effects not only on the filter performance of the bioreactors but also on the composition of the microbial communities. However, it is worth noting that BTF2 had a similar removal performance on day 88 compared to BTF1. This finding suggests that the structure of the microbial communities was heavily influenced by the operation mode, but their degradation functions were determined more by the composition of the waste gases.

*Burkholderia* and *Mycobacterium* were the most predominant genera in BTF1 and BTF2, respectively. The random forest model showed that they were also the two most important genera contributing to the differentiation of microbial communities between the two BTFs. The genus *Mycobacterium* and other members, of which relative abundance increased after the switch, together comprised the core microbial community of BTF2. Members of the genus *Mycobacterium* can retain viability under starvation conditions by entering a nonreplicating resting state (Gengenbacher et al. 2010; Wu et al. 2016). The degradation of toluene, ethyl benzene, and *o*-xylene by the genus *Mycobacterium* is also reported in the literature (Zhang et al. 2013). Additionally, the genus *Mycobacterium* predominated in BTF1 with relative abundances of 2.56% (first stage) and 1.88% (second stage). However, its relative abundance was far lower than the relative abundance of the genus *Burkholderia*. It is most likely that the growth rate of the genus *Mycobacterium* was lower than that of the genus *Burkholderia* when VOCs were continuously fed. This could be further confirmed by the fact that the relative abundance of the genus *Burkholderia* increased up to 10 times in BTF2 after the switch, while an increase of only 45% was observed for the genus *Mycobacterium*. Members of the genus *Burkholderia* are widely reported as having high degradation capabilities for numerous kinds of pollutants (Wang et al. 2002; Johnsen et al. 2005; Lee et al. 2016). However, unlike the genus *Mycobacterium*, some members of the genus *Burkholderia* may not be tolerant of the stress of starvation.

Biofilm formation is precisely regulated by a complex network involving multiple bacterial mechanisms such as quorum sensing and the two-component and c-di-GMP system (Coggan and Wolfgang 2012; Rasamiravaka et al. 2015). The metagenomics inferred by the PICRUSt method provided some helpful information for understanding the effect of operation mode on the ecological function of the microbial communities and on biofilm formation. Membrane transporters such as transporter, ABC transporter and secretion proteins are responsible for the transportation of a variety of substances across the cell membrane. Gene family encoding transporters were more abundant in BTF1 than those in BTF2, indicating that microorganisms grown inside BTF1 had high nutrient uptake rates. Hence, cell numbers increased to the threshold that the quorum sensing system required. Membrane transporters have reportedly played important roles during the biofilm formation of *Pseudomonas fluorescens* (Hinsa et al. 2003). The accumulation of exopolysaccharide was decreased significantly in a transporter-deficient mutant of *Rhizobium leguminosarum* compared to that of its wild type, and the mutant was severely impaired in terms of biofilm formation (Vanderlinde et al. 2010). Two-component signal transduction systems enable microorganisms, such as *Pseudomonas*, *Escherichia* and *Vibrio*, to regulate extracellular polysaccharide production and biofilm formation (Martinez and Vadyvaloo 2014; Rasamiravaka et al. 2015; Teschler et al. 2015). A review of the literature demonstrated that motility is a physiological and behavioral trait that is usually linked to the response to environmental gradients. The chemotactic response that is driven by environmental sensing and cell motility is important for microorganisms to search for available nutrients (Dang and Lovell 2016). The decrease in cell motility in BTF2 after the switch in the operation mode indicated a transition of microbial lifestyle from free-living forms to a biofilm.

Although PICRUSt revealed the changes in those gene families involving biofilm formation and pollutant degradation, it remains largely unknown to what degree the operation mode affected the biofilm formation. The determination of biomass showed that biofilm formation was greatly increased in BTF2 after the switch of operation mode, despite the fact that the relative abundance of the majority of these gene families decreased. In this sense, the quantitative analysis of the functional genes involved in biofilm formation is necessary. However, comparing all inferred gene families demonstrated that many gene families involved in the degradation of aromatic compounds and terpene series as well as the metabolism of amino acids and other carbohydrates were obviously upregulated in BTF2 compared to those in BTF1 after the switch in the operation mode. Linking this change to the microbial community in BTF2 could indicate that the genus *Mycobacterium* and the unclassified OTU 8121 may be the two most important degraders of aromatic compounds since their relative abundance dramatically increased after the switch in the operation mode.

## Abbreviations

BTF: biotrickling filter
VOCs: volatile organic compounds
EBRT: empty bed residence time
EPS: extracellular polymeric substances
GC: gas chromatograph
RE: removal efficiency
